# Supplementary material for: A survey of International Health Regulations National Focal Points experiences in carrying out their functions
Source: Global Health. 2021 Mar 6;17:25. doi: 10.1186/s12992-021-00675-7 (PMC7936598; doi:10.1186/s12992-021-00675-7)
Supplement: Supplementary file 1 — Additional file 1. [file 12992_2021_675_MOESM1_ESM.docx]

**Appendix 1**

|  | **Survey on the experiences of NFPs in implementing the IHR**  Welcome to the quantitative survey assessing the experiences and needs of NFPs in fulfilling their functions under the IHR.  For every question, please choose one of the options provided. For open ended text boxes, please insert your individual answers.  Your participation in this study is anonymous and results of individual countries will not be published. The website used for this survey is secure and ensures strict confidentiality. The study does not intend to test the knowledge or performance of NFPs. | |
| --- | --- | --- |
|  | Thank you very much – we appreciate the time and effort you have taken to complete this survey. The results of this study will be fed back to you in due course. | |
|  | Please choose one of the options provided | |
|  | **Please select your country** | |
|  | **Please choose a language, then press the "=>" button at the bottom of the page (English, French, Spanish, Russian, Chinese, Arabic)** | |
|  | **Please indicate how many years you work at the NFP office (<1, 2-3,3-4, 5-7, ≥8; select average numbers of years if the survey is completed by a group of NFP representatives)** | |
|  |  | |
| 1. **General Questions Regarding the Implementation of Mandatory IHR Functions:** 2. **Where is your NFP office located within the national governmental structure?** | |  |
|  | |  |
| Ministry of Health | |  |
| Ministry of Foreign Affairs | |  |
| Inter-ministerial body | |  |
| Other governmental entity (Please specify: __________________) | |  |
| 1. **To what extent are you familiar with the mandatory NFP functions under the IHR?** | |  |
| Not at all | |  |
| Somewhat | |  |
| Unsure | |  |
| Mostly | |  |
| Completely | |  |
| 1. **Do all ministries/agencies with responsibilities relevant to the implementation of the IHR know:** | |  |
| - 1. The role of the NFP? | |  |
| All | |  |
| Most | |  |
| Some | |  |
| Few | |  |
| None | |  |
| - 1. How and when to engage with the NFP? | |  |
| All | |  |
| Most | |  |
| Some | |  |
| Few | |  |
| None | |  |
| 1. **Accessibility and Communications with WHO Regional IHR Contact Points** | |  |
| 1. **Does your NFP know how to contact the designated WHO Regional IHR Contact Point?** | |  |
| Yes | |  |
| No | |  |
| 1. **Does your NFP have the necessary content expertise to discuss a notifiable event with the WHO Regional IHR Contact Point?** | |  |
| Yes | |  |
| No | |  |
| 1. **Do you have a system in place to ensure the NFP is accessible at all times (24/7/365) for urgent communications to WHO concerning the implementation of the IHR?** | |  |
| Yes | |  |
| No | |  |
| 1. **Does your NFP have adequate information technology (IT) systems in place to carry out NFP communication functions?** | |  |
| Yes | |  |
| No | |  |
| 1. **To what extent do you agree with the following statements regarding the ability of your NFP to carry out functions as detailed in Article 4 of the IHR:** | |  |
| 1. **Our NFP is able to send to WHO, on behalf of the State Party, urgent event-related communications under the IHR (e.g., notifications under Article 6 of the IHR).** | |  |
| Strongly Agree | |  |
| Somewhat Agree | |  |
| Unsure | |  |
| Somewhat Disagree | |  |
| Strongly Disagree | |  |
| 1. **Our NFP is able to disseminate information from WHO to relevant sectors of the State Party and consolidate input from these sectors in a timely fashion.** | |  |
| Strongly Agree | |  |
| Somewhat Agree | |  |
| Unsure | |  |
| Somewhat Disagree | |  |
| Strongly Disagree | |  |
| 1. **How much do the following impact your timely notification of a public health event to WHO? (1 – no impact, 5 – major impact).** | |  |
| NA – No notifiable events have occurred | |  |
| - 1. **Concern about impact on tourism and/or trade** | |  |
| 1 | |  |
| 2 | |  |
| 3 | |  |
| 4 | |  |
| 5  N/A | |  |
| - 1. **Political challenges within your country** | |  |
| 1 | |  |
| 2 | |  |
| 3 | |  |
| 4 | |  |
| 5  N/A | |  |
| - 1. **Lack of NFP authority** | |  |
| 1 | |  |
| 2 | |  |
| 3 | |  |
| 4 | |  |
| 5  N/A | |  |
| - 1. **Insufficient human resources** | |  |
| 1 | |  |
| 2 | |  |
| 3 | |  |
| 4 | |  |
| 5  N/A | |  |
| - 1. **Insufficient financial resources** | |  |
| 1 | |  |
| 2 | |  |
| 3 | |  |
| 4 | |  |
| 5  N/A | |  |
| - 1. **Lack of necessary legal authority or arrangement** | |  |
| 1 | |  |
| 2 | |  |
| 3 | |  |
| 4 | |  |
| 5  N/A | |  |
| - 1. **Limited resources in the country to carry out surveillance/detection of events** | |  |
| 1 | |  |
| 2 | |  |
| 3 | |  |
| 4 | |  |
| 5  N/A | |  |
| - 1. **Uncertainty over how to notify/report an event to WHO** | |  |
| 1 | |  |
| 2 | |  |
| 3 | |  |
| 4 | |  |
| 5  N/A | |  |
| - 1. **Concern about confidentiality of information sent to the WHO** | |  |
| 1 | |  |
| 2 | |  |
| 3 | |  |
| 4 | |  |
| 5  N/A | |  |
| - 1. **Concern about how the WHO will use the information that is reported to them (e.g., dissemination of information to other countries).** | |  |
| 1 | |  |
| 2 | |  |
| 3 | |  |
| 4 | |  |
| 5  N/A | |  |
| - 1. **Concern about damage to the country’s image (i.e., reputational risk)** | |  |
| 1 | |  |
| 2 | |  |
| 3 | |  |
| 4 | |  |
| 5  N/A | |  |
| - 1. **Please provide any additional information about how these factors have affected your ability to report notifiable events: __________________** | |  |
| 1. **Has there ever been a time(s) when your NFP did not notify an event to WHO that met at least two of the four criteria in the IHR Annex 2 decision instrument?** | |  |
|  | |  |
| Yes | |  |
| No | |  |
| If yes, why did you not report on this occasion _______________ | |  |
| 1. **The last time you notified an event to WHO, to what extent was the decision to notify WHO based on the criteria outlined in the IHR Annex 2 decision instrument?** | |  |
|  | |  |
| Not at all | |  |
| Somewhat | |  |
| Unsure | |  |
| Mostly | |  |
| Completely | |  |
| Have never notified WHO | |  |
| 1. **The last time you reported an event to WHO (notification or consultation), to what extent did you receive adequate support to help respond to the event from:** | |  |
| - 1. **WHO** | |  |
| Not at all | |  |
| Somewhat | |  |
| Unsure | |  |
| Mostly | |  |
| Completely | |  |
| NA | |  |
| - 1. **Your national government** | |  |
| Not at all | |  |
| Somewhat | |  |
| Unsure | |  |
| Mostly | |  |
| Completely | |  |
| NA | |  |
| - 1. **Other relevant stakeholders (public agencies/local authorities)?** | |  |
| Not at all | |  |
| Somewhat | |  |
| Unsure | |  |
| Mostly | |  |
| Completely | |  |
| NA | |  |
| 1. **How often does your NFP communicate bilaterally with other countries’ NFPs for assistance/guidance in carrying out its functions or exchange of information?** | |  |
| Often | |  |
| Sometimes | |  |
| Rarely | |  |
| Never | |  |
| 1. **If a peer-to-peer NFP network was developed, do you believe this network should be:** | |  |
| Officially overseen by WHO | |  |
| Independent of WHO | |  |
| 1. **Disseminating Information to and Consolidating Information from Relevant Sectors regarding Urgent Communications** | |  |
| 1. **Thinking about the last time you reported an event to WHO under the IHR, were there procedures and structures in place for timely communication between the NFP and the relevant sectors/bodies at the national level?** | |  |
| Yes | |  |
| No | |  |
| Have not reported to WHO | |  |
| 1. **Thinking about the last time you reported an event to WHO under the IHR, did your NFP:** | |  |
| - 1. **Have access to the necessary ministries and decision-makers, including senior management?** | |  |
| Yes | |  |
| No | |  |
| Have not reported to WHO | |  |
| - 1. **Require clearance from other sectors before an event can be notified to WHO?** | |  |
| Yes | |  |
| No | |  |
| Have not reported to WHO | |  |
| 1. **How long does it usually take from the start of your notification assessment until the actual notification to WHO, if an event is deemed notifiable?** | |  |
| 24-48h | |  |
| 48-72h | |  |
| >72h | |  |
| 1. **Overall NFP Functions** | |  |
| 1. **Do you feel there is a role for mobile or information technology to improve your NFP’s ability to fulfill its functions under the IHR (e.g., applications to facilitate the process of getting approvals and reporting to WHO under the IHR)?** | |  |
| Yes | |  |
| No | |  |
| If yes, how? _____________ | |  |
| 1. **To what extent are you familiar with the optional NFP functions as outlined in the Appendix of WHO’s NFP Guide?** | |  |
| Not at all | |  |
| Somewhat | |  |
| Unsure | |  |
| Mostly | |  |
| Completely | |  |
| 1. **Is there a plan being implemented at your NFP office to support the continuous development and learning of staff working in the NFP?** | |  |
| Yes | |  |
| No | |  |
| 1. **WHO Guidance Documents and Training Tools** | |  |
| 1. **How useful do you find:** | |  |
| - 1. **WHO’s NFP Guide?** | |  |
| Very Useful | |  |
| Somewhat Useful | |  |
| Not Very Useful | |  |
| Not Useful at All | |  |
| Not Aware of this Tool | |  |
| - 1. **Annex 2 Tutorials?** | |  |
| Very Useful | |  |
| Somewhat Useful | |  |
| Not Very Useful | |  |
| Not Useful at All | |  |
| Not Aware of this Tool | |  |
| - 1. **WHO’s “Toolkit for Implementation in National Legislation”?** | |  |
| Very Useful | |  |
| Somewhat Useful | |  |
| Not Very Useful | |  |
| Not Useful at All | |  |
| Not Aware of this Tool | |  |
| - 1. **IHR training toolkit online course?** | |  |
| Very Useful | |  |
| Somewhat Useful | |  |
| Not Very Useful | |  |
| Not Useful at All | |  |
| Not Aware of this Tool | |  |
| - 1. **Global or Regional NFP knowledge network(s)?** | |  |
| Very Useful | |  |
| Somewhat Useful | |  |
| Not Very Useful | |  |
| Not Useful at All | |  |
| Not Aware of this Tool | |  |
| - 1. **NFP workshops organized by the WHO Regional Office?** | |  |
| Very Useful | |  |
| Somewhat Useful | |  |
| Not Very Useful | |  |
| Not Useful at All | |  |
| Not Aware of this Tool | |  |
| - 1. **Simulation exercises organized by WHE (e.g., JADE)?** | |  |
| Very Useful | |  |
| Somewhat Useful | |  |
| Not Very Useful | |  |
| Not Useful at All | |  |
| Not Aware of this Tool | |  |
| - 1. **Please provide any additional information about how these factors have affected your ability to report notifiable events: __________________** | |  |
| 1. **Can you think of any new elements of guidance that should be included in a revised version of WHO’s NFP Guide?** | |  |
| Please list up to five most useful additions WHO could make to the NFP Guide: **__________________________** | |  |
| 1. **In your opinion, what are the three most useful additions WHO could make to the NFP training process in order of importance? __________________________** | |  |
| 1. **In your opinion, what are the three most useful contributions WHO could provide to support NFP in fulfilling their mandatory functions under the IHR? __________** | |  |
| 1. **Are the WHO training tools available to you in your preferred language?** | |  |
| Yes | |  |
| No | |  |
| 1. **Please rank the five most effective and user-friendly training and guidance tools to support the functioning of your NFP: (5 highest and 1 lowest)** | |  |
| computer based online courses 1  2  3  4  5 | |  |
| mobile learning for more ready access (mobile phone, tablet) 1  2  3  4  5 | |  |
| standard operating procedures that could be customized for your NFP 1  2  3  4  5 | |  |
| rapid learning modules (condensed, shorter training tools) 1  2  3  4  5 | |  |
| webinars with experts 1  2  3  4  5 | |  |
| sharing of experiences (platform for bilateral communication amongst NFPs and sharing of materials and learnings) 1  2  3  4  5 | |  |
|  | peer-to-peer learning 1  2  3  4  5 | |
| coaching/mentoring 1  2  3  4 5 | |  |
| face-to-face group discussions 1  2  3  4 5 | |  |
| 1. virtual group discussions   1  2  3  4 5 | |  |
| readings 1  2  3  4 5 | |  |
| lectures 1  2  3  4 5 | |  |
| videos 1  2  3  4 5 | |  |
| case studies 1  2  3  4 5 | |  |
| 1. simulation exercises   1  2  3  4  5 | |  |
| 1. **Can you think of any other learning materials and training tools that may be useful for your NFP?**   **__________________________** | |  |
| 1. **Have you developed any learning materials and training tools for your NFP office (e.g. SOPs, best practices, orientation courses, etc.)?**   **__________________________** | |  |
| 1. **Would you like to share those materials and tools with WHO and other NFPs? If yes, please upload or enter the relevant URL:**   **__________________________** | |  |
|  | |  |
